# Optimal Path Routing Protocol for Warning Messages Dissemination for Highway VANET

**DOI:** 10.3390/s22186839

**Published:** 2022-09-09

**Authors:** Mumtaz Ali Shah, Farrukh Zeeshan Khan, Ghulam Abbas, Ziaul Haq Abbas, Jehad Ali, Sumayh S. Aljameel, Irfan Ullah Khan, Nida Aslam

**Affiliations:** 1 Department of Computer Science, University of Engineering and Technology, Taxila 47080, Pakistan; 2 Telecommunications and Networking Research Center, GIK Institute of Engineering Sciences and Technology, Topi 23640, Pakistan; 3 Faculty of Computer Science and Engineering, Ghulam Ishaq Khan (GIK) Institute of Engineering Sciences and Technology, Topi 23460, Pakistan; 4 Faculty of Electrical Engineering, GIK Institute of Engineering Sciences and Technology, Topi 23460, Pakistan; 5 Department of Computer Science and Engineering, Sejong University, Seoul 05006, Korea; 6 Department of Computer Science, College of Computer Science and Information Technology, Imam Abdulrahman Bin Faisal University, P.O. Box 1982, Dammam 31441, Saudi Arabia; 7 SAUDI ARAMCO Cybersecurity Chair, College of Computer Science and Information Technology, Imam Abdulrahman Bin Faisal University, P.O. Box 1982, Dammam 31441, Saudi Arabia

**Keywords:** vehicular ad hoc networks, cluster stability, warning messages, bi-directional road traffic, routing protocol, security, safety application

## Abstract

In vehicular ad hoc networks (VANETs), helpful information dissemination establishes the foundation of communication. One of the significant difficulties in developing a successful dissemination system for VANETs is avoiding traffic fatalities. Another essential success metric is the transfer of reliable and secure warning messages through the shortest path, particularly on highways with high mobility. Clustering vehicles is a general solution to these challenges, as it allows warning alerts to be re-broadcast to nearby clusters by fewer vehicles. Hence, trustworthy cluster head (CH) selections are critical to decreasing the number of retransmissions. In this context, we suggest a clustering technique called Optimal Path Routing Protocol for Warning Messages (OPRP) for dissemination in highway VANETs. OPRP relies on mobility measured to reinforce cluster creation, evade transmission overhead, and sustain message authenticity in a high mobility environment. Moreover, we consider communication between the cluster heads to reduce the number of transmissions. Furthermore, the cluster head is chosen using the median technique based on an odd or even number of vehicles for a stable and lengthy cluster life. By altering traffic densities and speeds, OPRP is compared with prominent schemes. Simulation results revealed that OPRP offers enhanced throughput, end-to-end delay, maximizing packet delivery ratio, and message validity.

## 1. Introduction

**Figure 1:**
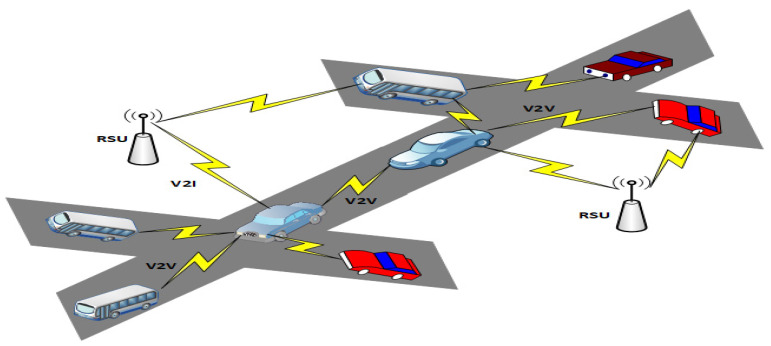
The general architecture of a VANET.

According to World Health Organization research [1], road accidents claim the lives of almost 1.3 million people every year around the world. VANETs offer a viable approach for avoiding such incidents. VANETs are mobile ad hoc networks [2] that can operate with a diversity of models, such as the Vehicle-to-Infrastructure (V2I) model and Vehicle-to-Vehicle (V2V) model, as depicted in Figure 1. Advanced communication protocols such as IEEE 802.11p and IEEE 1609 send information between vehicles in the fundamental VANET architecture. VANETs encompass a diverse set of utilizations divided into two categories, i.e., comfort and safety applications. Cooperative collision prevention and traffic updates are examples of safety applications.

In contrast, comfort applications provide value-added services, including entertainment, travel time prediction, path identification, environmental protection, and road conditions [3]. The prevention of traffic accidents is the most crucial application among these. Safety applications in VANETs mostly rely on warning message (WM) broadcasting. When a vehicle senses a hazardous incident, it transmits WMs to the surrounding vehicles, allowing them to take appropriate action to avoid traffic accidents [4,5]. However, on the other hand, unnecessary broadcasting causes several serious problems, including road accidents, redundancy in transmission, and high latency [6].

Therefore, a rapid and reliable path for WMs is critical, as latency and packet drops during WMs delivery can cause large-scale collisions among vehicles [6]. When a sender vehicle is ready to send WMs, it may face two scenarios. To begin with, if there is just one path to the target vehicle, it is unavoidable to take that path. Secondly, choosing the optimal route with the least latency and packet drop becomes critical if multiple paths to the target vehicle are available. As a result, selecting the following hop, also known as the optimal forwarder (*Of*), is critical during the delivery of WMs.

Greedy routing methods [7] consider parameters such as the present positions of intermediate relay nodes and their distances from the target node. When vehicles are static, such parameters are valid. In VANETs, the greedy routing protocols [8] chose the next-hop in a target vehicle direction, which is a good strategy in uni-directional road traffic. However, direction-based greedy protocols’ performances worsen in bi-directional road traffic when a next-hop is picked based on its current location in the direction of the target vehicle. The distances among vehicles change in a bi-directional highway environment because nodes can move at high speeds in both directions. Hence, due to the constant changes in topology, these vehicles leave and enter the transmission ranges with one other regularly. As a result, a path chosen at a specific time step does not remain fixed indefinitely. It may alter at later time-steps somewhat for transmitting even a single warning message. This alteration may happen concerning a decreased or increased number of hops that affect the latency appropriately. Furthermore, paths are broken, and new paths are generated at routine intervals, potentially resulting in network partitions. Therefore, all warning messages routed across the damaged paths are dropped. These aspects lead to high packet losses, delays in WMs transmission, and reduced throughput of the networks.

We present a unique cluster-based approach, OPRP, to disseminate WMs in highway VANETs, addressing the abovementioned difficulties. OPRP considers our mobility parameters when choosing a CH to improve cluster stability and reduce communication overhead. Only a cluster head can disseminate the WMs among its cluster members. Moreover, CH transmits WMs across adjacent cluster heads and increases network efficiency.

### Novelty and Contributions

We used a hybrid mechanism to disseminate warning messages. For this, we considered a cluster-based bi-directional highway scenario. The contributions and novelty are as follows:

- Dissemination of warning messages has been performed on bi-directional highway roads, but in a non-cluster scenario. The novelty of this paper is the directional-based clustering technique for disseminating warning messages on bi-directional highways.
- We propose a modified K-medoids algorithm for selecting cluster heads. To the best of our knowledge, the modified K-medoids have never been used in the literature for cluster head selection in a bi-directional highway scenario. The modified K-medoids algorithm is presented in Section 3.2.3, Equation (5).
- We use the positions and movement directions of the nodes to disseminate warning messages in a clustering-based approach. Previously, either the positions or movement directions of nodes have been used, but not both.
- We use the Hamming distance function to find the direction of a node. To the best of our knowledge, Hamming distance has never been used previously in a bi-directional clustering-based approach. It is presented in Section 3.2.1, Equation (2).
- We use Euclidean distance for cluster formation in the bi-directional highway. To the best of our knowledge, this was the first time Euclidean distance was employed for cluster formation in a bi-directional clustering-based approach.

Simulation results reveal that OPRP outperforms renowned WMs dissemination schemes concerning information coverage, end-to-end delay (E2E), network overhead, message reliability, and PDR. The remainder of the paper is organized as follows: Section 2 reviews related work. Section 3 offers a system model, and Section 4 estimates its performance. Finally, Section 5 presents the conclusion and future work.

## 2. Related Work

The dissemination of WMs in VANETs has received a lot of interest in recent years. By broadcasting WMs on time, drivers can avoid collisions. As a result, dissemination speed is a vital performance component, particularly for time critical WMs [9]. Designing such solutions for enormously dynamic vehicle networks is extremely difficult. Therefore, timely WMs transmission and computationally effective solutions are required for WM dissemination in VANETs. The schemes discussed in this subsection address specific WM dissemination issues, such as lowering network overhead and end-to-end delay while boosting information coverage and PDR.

In VANETs, timely and reliable warning messages are as critical as accurate collision detection. For VANETs, several clustering techniques have been suggested. The authors of [10] presented a clustering technique for VANETs called the Distributed Multichannel and Mobility-Aware Cluster-based MAC Protocol (DMMAC). The DMMAC considers speeding a significant aspect while forming the cluster to improve stability, using a fuzzy framework to measure vehicle speeds. Furthermore, the DMMAC uses a temporary cluster head idea when the original cluster head is unreachable. For every cluster, the protocol chooses a secondary CH. These protocols [10,11] are helpful in high-mobility areas, although primary cluster heads frequently change as topologies change. As a result of the frequent switching generated by the primary CH, the secondary cluster head’s efficiency is diminished. Therefore, the clusters become unstable.

Furthermore, the authors of [12] offered a novel clustering approach that relies on affinity propagation to tackle instability. This technique does not need a constant number of nodes to create a cluster; instead, it employs similarity measurements to transmit messages across data points throughout cluster creation. Although the affinity technique can aid with cluster stability, it does so by involving several iterative loops, which lengthens cluster creation time, and as a result, produces more significant latency.

To achieve effective bandwidth utilization and minimize message delivery time [13] introduced an event-driven cluster-based method. Furthermore, clustering after event finding produces an end-to-end delay, which is inconvenient for time-crucial information in bi-directional road traffic. The authors of [14] suggested a clustering strategy called time barrier-based emergency message dissemination in the vehicular ad-hoc network (TBEM) that relies on the time barrier technique and aims to reduce excessive message dissemination. If an event occurs during work, the farthest vehicle is a relay to cover a greater distance. As there are multiple vehicles along the same length of road, numerous vehicles can send the same message, causing network congestion. Furthermore, vehicles are permitted to transmit messages after the time limit expires, resulting in redundancy and affecting network performance.

The authors of [15] suggested the distributed vehicular broadcast (DV-CAST) protocol to enhance coverage area and eliminate detached network difficulties adaptively. DV-CAST is the only solution we identified in the literature that tackles varying connectivity situations in VANETs. To improve coverage information, DV-CAST uses a store-carry-forward (SCF) method [16,17,18,19]. Furthermore, the transmitting vehicles send WMs to the farthest vehicle with a high probability of shortening the waiting time. However, when the distance between two vehicles grows, a message’s transmission probability increases linearly. As a result, the SCF method causes end-to-end latency. Multiple vehicles can retransmit the same message in a probabilistic approach, causing network congestion.

The k-medoids [20] and k-means [21] algorithms add to the clustering diversity but have similar tendencies. However, a k-medoids algorithm performs better in some cases [22]. Both algorithms divide the entire sample space into groups of comparable nodes depending on the shortest distance among nodes and a central node, CH. In a k-medoids algorithm, the cluster’s center is always a node, but in a k-means algorithm, it may or may not be. As the mobility of nodes in VANET is high, choosing a geographical place other than the node as the cluster center can induce clustering instability. Furthermore, any approximation made in this direction by choosing the closest node to a central place will reduce accuracy and increase processing overhead. Moreover, compared to a k-means algorithm, a k-medoids algorithm is much more tolerating of outliers [21].

The study in [23] presents the fuzzy logic-based technique for creating clusters with long lifetimes. Three parameters are taken into account in this approach. First, it combines node relative speeds with their associated CHs. Second, it examines a node’s number of available links inside a cluster, and third, it considers node security. This method can still be used in uni-directional highway scenarios. In a bi-directional highway scenario, however, selecting member nodes with the same speed as its CHs may be ineffective because they could travel in different directions. This reduces the time it takes for the node to be associated with a cluster. Another similar clustering strategy was proposed in [24]; however, it lacks the potential to tolerate the variability of bi-directional road traffic. In addition, the authors of [25] considered connection dependability during clustering. However, this strategy assumes a fixed arrival rate for motorway nodes, which is unsustainable.

The work of [26] presents a comparative distance-based clustering algorithm that performs poorly in real highway scenarios due to a lack of direction evaluation. Likewise, the authors of [27] adopted a grey wolf optimization-based clustering method to limit the number of clusters. On the other hand, the approach has a significant computational overhead when identifying weak and aging nodes. One well-known clustering protocol is Low-Energy Adaptive Clustering Hierarchy (LEACH) [28], which has various multi-hop and single-hop variations. LEACH Fuzzy Inference System (LEACH-FIS) [29] and Quadrature Low Energy Adaptive Cluster Hierarchy (FCM-QLEACH) [30] are two current VANET-specific variations. All LEACH techniques are energy-efficient, which is their main advantage, but significant clustering overheads limit their performance.

Similarly, the authors of [31] presented a clustering technique in which a gateway node is introduced as a relay node between CHs. The gateway node provides the connection between two cluster heads to increase information range without needing roadside units. This scheme is best for uni-directional traffic, and suitable for urban VANETs and unsuitable for highway environments. EEMDS also ignores the node’s direction, degrading the scheme’s performance. The work of RBO-EM [32] presents a clustering scheme in which the nodes’ numbers are reduced that can retransmit an emergency message, and linked condition reliability is employed to choose a trustworthy gateway. RBO-EM also ignores the node’s direction, degrading the scheme’s performance.

Similarly, the authors of BRUP [33] presented a network that was hierarchically partitioned into numerous clusters on a roundabout in an urban scenario, each associated with the CH. Only the CH was accountable for the retransmission of WMs in every cluster to avoid redundant transmission and ensure reliable WM dissemination. Furthermore, ref. [33] employed a k-medoids algorithm for CH selection and Hamming distance for finding a node’s movement direction to maximize the cluster’s lifetime, and it has been proven to operate well in a roundabout in urban settings. BURP does not apply to highways environment where mobility is very high. This paper proposes OPRP as an extension of [33] to highway environments. The authors of [1,34] described message dissemination using SND. However, SDN separates the data and control plane, and the controller should interact with the underlying network to have a global view of the status of the data plane. Hence, when there are dynamic changes in the network, there should be a frequent status update in the SDN controller.

When there is a new packet for which the flow rules do not exist in the underlying switches, it must be sent to the controller, and the same applies for the updated status. Consequently, it adds delay. Moreover, when the algorithm for dissemination runs and the new updates rules are found, those rules must be pushed to the flow tables of the SDN switches. Hence, there will be an additional flow setup delay. Table 1 shows a comparison of several WM dissemination techniques.

## 3. Proposed Protocol

This section proposes our protocol (OPRP) enabling V2V communication betwixt nodes traveling in the real-time bi-directional highway environment. The timely propagation of WMs is crucial in VANET environments, necessitating an effective routing mechanism. In the following subsection, we propose the system model for bi-directional highway VANETs. CH selection, WM distribution, and dynamic cluster formation are all included in the proposed approach.

### 3.1. System Model

**Figure 2:**
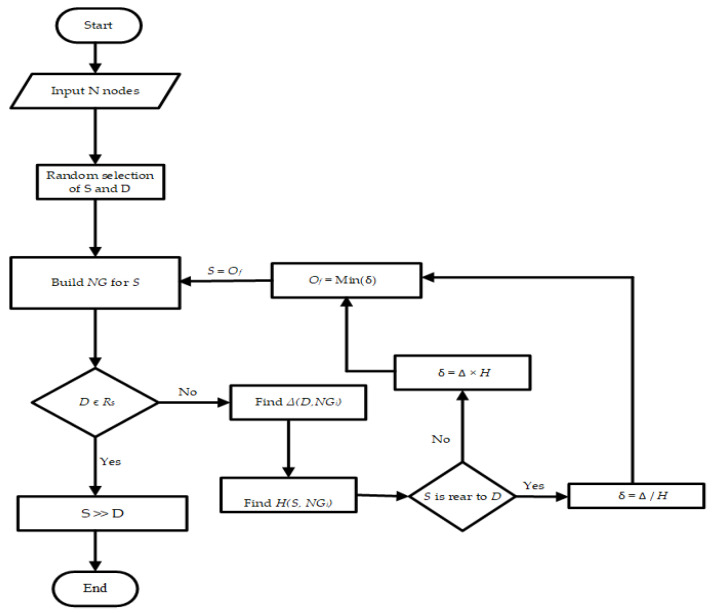
An overview of the OPRP.

Figure 2 depicts a detailed outline of the proposed protocol, which achieves direction-based priority assignments among numerous routes to a destination. Collisions among nodes on a wide scale could be caused by an ineffective routing strategy for vehicle collision prevention. As a result, routing performance becomes equally essential in addition to the importance of node collision detection. Delivering timely and reliable WMs is crucial for implementing collision-prevention procedures. Once a source node is willing to send a WM, and there is only one path to the destination, following that path becomes unavoidable. When there are numerous paths to the target, choosing the optimum path with the least latency, the fewest packet losses, and the highest throughput becomes critical. Greedy protocols use distance as a parameter to find the optimum path, whereas direction-based Greedy protocols look for available relay nodes in the direction of the destination node. All paths to the target are examined for a specified time step, such as τ0. However, the node’s mobility is very high; they regularly enter and exit each other’s communication ranges, resulting in continual topological changes in bi-direction circumstances. Due to this dynamic nature, two scenarios may arise during message delivery.

Firstly, paths to the target may encounter outages, causing all communications transmitted on these paths to be lost. Second, because the path specified at τ_0_ does not always stay intact, it may go through a path reconstruction phase at τ_1_, τ_2_, …, τ_n_ until a message is successfully delivered.

This might be increasing or decreasing the length of a path. As a result, the rate of packet drops and delays increase, reducing the throughput of the Greedy and direction-based Greedy protocols. Therefore, in complement to a distance parameter, we recommend using the following parameters for the optimum path selection:

- The direction of node movement: here, we apply Equation (1) to find the direction of a node’s movement.
- The source and destination nodes’ relative positions.
- The list of notations used in this research can also be found in Table 2. In this regard, the proposed protocol includes two phases: the cluster formation phase and the optimal path discovery phase. The subsections below go over all of these phases in detail.

### 3.2. Cluster Formation Phase

The following assumptions are taken into account by OPRP:

- Each node starts in a safe state, with no probability of crashing.
- Each node has the on-board unit (OBU) for communication and a Global Positioning System (GPS) for positioning.
- Each node can determine its direction, speed, location, and deceleration or acceleration.
- Each node may calculate its maximum average radio range based on its surroundings, which can be utilized to connect and communicate with the other nodes.

Ordinary vehicles (OV) and CHs are the two types of nodes that the suggested OPRP applies to. A CH is in charge of the cluster and keeps a record of all nodes that make up the cluster. An OV represents a non-CH member node. A CH constructs a list of each of its member nodes, which includes current location, current speed, node-id, expected state, and acceleration/deceleration. The following are possible states for a node:

- The node moves forward from a standstill to enter the highway. In this circumstance, it will either join an established cluster or establish its cluster.
- The node maintains the same direction and speed on the highway. In this situation, it continues to move with the same cluster. During the travels, its speed may alter as it accelerates or decelerates with time, resulting in a change in its cluster.
- While moving with the highway, a node may reverse its heading and begin moving in the opposite direction. This will result in a cluster change for this particular node.

This subsection of the suggested OPRP describes cluster creation, including how nodes can find their neighbors and enter or depart a cluster. The k-medoids algorithm is possibly a better option for clustering based on the benefits stated in the previous section. The simple k-medoids approach, on the other hand, is insufficient for clustering bi-directional road traffic. The direction parameter is considered to accommodate bi-directional road traffic. To that end, in the suggested OPRP, we present a variation to the fundamental k-medoids algorithm and employ the modified form for clustering. The rest of this section explains the various phases required in clustering in a bi-directional environment using the revised k-medoids algorithm.

#### 3.2.1. Neighbor Discovery

We offer Algorithm 1, which takes *Ack* and *N* as inputs and finds neighbors, where *N =* *n_1_,* *n_2_,* *…,* *n_x_* denotes a set of all nodes, and *Ack* denotes an acknowledgment received in response to a transmitted Hello packet. Here, *n* denotes a single member node, and x represents the number of nodes on the path. The algorithm discovers neighbor nodes and creates a neighboring table (*NG*), which is a list of all of a node’s neighbors. Neighbors are nodes connected directly to one another or within one hop. Each node broadcasts Hello packets at regular intervals to discover its neighbors.
**Algorithm1:** Neighbor Discovery.**Input:** *Ack,N***Output:** *NG*  1.**Begin:**  2.**Flush** *NG_s_*  3.*i*←0  4.*S* broadcast *Hello* packets  5.             **Repeat:**  6.                  for A*ck* received from node *n_i_*  7.                  *S* updates the *NGs*  8.                      *NGs* (*i*, 1)←*NID_ni_*  9.                      *NGs* (*i*, 2)←*CNP_ni_*  10.                     *NGs* (*i*, 3)←𝜏*_ni_*  11.                     **increment** ***i***
  12.              **until:** All the received *Acks* are processed or each cluster  13.   **Return** *NG*  14.**end**

When S obtains an Ack from another node in reply to its broadcasted Hello packet, it adds this node to its *NG*. In *NG*, information about nearby nodes is maintained in the form of current node position (*CNP*), node identity (NID), and the time stamp (𝜏) of the last correctly received *Ack*. Here, 𝜏 is used to determine the freshness of the received Ack packet and to remove any older information. For node localization, we suppose every node is fitted with a Global Positioning System (GPS). Algorithm 1 produces *NG* as a result. The algorithm’s complexity is on average Θ(n). The output of the algorithm is *NG* for the neighbors at each time period.

#### 3.2.2. Node Joining a Cluster

When different nodes come in, a highway becomes crowded with clusters. The highway’s first node forms its cluster and deputizes itself as a CH. The CH is re-selected for every cluster when the number of nodes within cluster grows, as explained in Section 3.2.3. A candidate node, a node that wants to join the cluster, sends out a Hello message. The OVs ignore this communication, but all CHs in the vicinity acknowledge it.

The number of acknowledgments a candidate node receives is the number of available clusters (k) for the k-medoids algorithm. The distance betwixt this candidate node and every CH is calculated when the position information is shared. The nearest CH is found, so this applicant node joins that cluster to which it belongs. Suppose a candidate node cannot find a cluster—i.e., it receives no acknowledgment in reply to a Hello message. The candidate node builds its cluster and deputizes itself as the CH.

The main k-medoids algorithm [20] uses Manhattan distance for clustering. In contrast, the modified k-medoids algorithm presented in this paper for OPRP employs two metrics, Euclidean distance, and Hamming distance, to create clusters. The first metric, Euclidean distance, calculates the distance between a candidate node and all of the CHs nearby. Hence,

(1) $$\eta \left(x,y\right)=\sqrt{{\left(x_{1}-x_{2}\right)}^{2}+{\left(y_{1}-y_{2}\right)}^{2}},$$

where *η* denotes the Euclidean distance calculated between the CHs and applicant node with coordinates ($x_{1}$,$\;y_{1}$) and ($x_{2}$,$\;y_{2}$), respectively.

The Hamming distance function, which includes the bi-directional aspect of a road traffic flow, is the next metric computed after the Euclidean distance. The nodes on the highway can move in opposite directions because the bi-directional heterogeneous traffic allows it. Hamming distance function calculates in light of the difference in travel directions between an applicant node and a CH. The result is 1 if a candidate node and the CH have the same direction and 0 if they have the opposite direction [33]; i.e.,

(2) $$\kappa =\left\{\begin{array}{cc} 1 & if\;=\;\mathrm{same}\;\\ 0 & if=\;\mathrm{opposite}, \end{array}\right.$$

here, the Hamming distance is denoted by κ, and the direction is represented by regardless of the node type (i.e., OV or CH).

**Figure 3:**
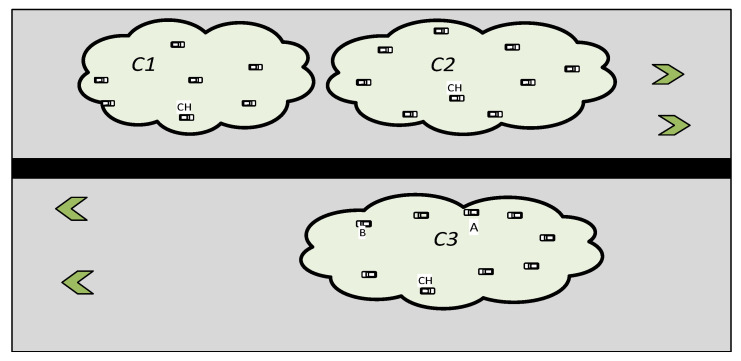
Cluster formation in bi-directional.

It is essential to rely on the two metrics described above when connecting nodes to a cluster. Assume, for instance, that Node A is located 3 m from C2: CH and 4 m from C3: CH in Figure 3. This node will connect to C2 using simply the distance factor. This should not occur, since Node A and the members of C2 are traveling in opposing directions, and Node A joining C2 would destabilize the cluster rather than serve a proper function. OPRP suggests Equation (3) to solve this problem, which determines the ultimate distance by adding the directional components and relative components. By applying this criterion, the distance between Node A and C2: CH increases to infinity while the distance between Node A and C3: CH stays at 4 m. As a result, Node A will join C3. As a result, by associating the node with the cluster in the same direction, a node equation used in a modified k-medoids algorithm for OPRP favorably and efficiently contributes to the definition of stable clusters. Hence,

(3) $$\delta =\frac{\eta }{\kappa }$$

where *δ* stands for the relative distance between two adjacent nodes.

The distance with all neighboring CHs is calculated by Equation (3) because OPRP considers the attachment of the candidate node to the cluster upon the shortest distance from the appropriate CH. Additionally, Equation (4) locates a CH with the smallest distance and links the node to the relevant cluster, converting that candidate node’s position to that of the member node. Thus,

(4) $$CH_{min}=\mathrm{Min}\left[\delta \left(S_{i},CH_{i}\right)\right],$$

where $CH_{min}$ is the minimum distance *CH*, $S_{i}\;$ represents the *i*th candidate node, and *CH_i_* is the *i*th cluster in the sample space.

Using our modified k-medoids technique, Algorithm 2 outlines how a node might join a specific cluster. The inputs for this algorithm are *k*, *M*, and *S*. *k* is the number of the clusters obtained in response to a Hello message, *M* is the set of CHs, and *S* is the set of applicant nodes. The algorithm’s output consists of *k* clusters created or modified by adding *N* nodes, where *N* stands for the collection of member nodes in the cluster. The algorithm’s complexity is on average Θ(n). The output of the algorithm is *K* clusters each time.
**Algorithm2:** Candidate Node Joining a Cluster.**Input:** *K*, *M*, *S***Output:** *K* cluster with *N* nodes  1.**Begin:**  2.**repeat:**  3.   **if** *K* ≠ 0 **then**  4.     **for** each member of *M* **do**  5.       Compute *η_i_* using (1)  6.       Compute *κ_i_* using (2)  7.       Compute δ*_i_* using (3)  8.      *T_i_* ⇐ δ*_i_*  9.      *CH_min_* ⇐ Min [T] using (4)  10.      *CH_min_* ← S*_i_*  11.      *N_min_* ← S*_i_*  12.     **end** **for**  13.    **else**  14.      *K* ⇐ *K* + 1  15.      *CH_i_* ← *S_i_*  16.      *M_i_* ← *CH_i_*  17.     **end** **if**  18.**until:** All the members of *S* are clustered  19.**end**

#### 3.2.3. Cluster Head Selection

The CH node in our suggested approach is the one that is the closest to every other node in the cluster. It is reducing the number of hops lowers communication latency. As seen in Figure 2, the CH selection procedure continues to iterate until the clusters merge. This convergence is best attained if no modification is seen after two iterations [20]. When a first potential node enters a highway and forms its own cluster, as was covered in the previous subsection, the CH is chosen. The modified k-medoids algorithm determines the median of each cluster when the clusters are filled with nodes. Therefore, this median, also known as the medoid, computed using Equation (5), is elected as the new CH for a cluster [33]. Thus,

(5) $$M_{c}=\left\{\begin{array}{cc} {\left[\frac{n+1}{2}\right]}^{th} & \mathrm{if}\;\mathrm{nodes}\;=\;\mathrm{odd}\;\\ {\left[\frac{n}{2}+1\right]}^{th} & \mathrm{if}\;\mathrm{nodes}\;=\;\mathrm{even}\; \end{array}\right.$$

where *Mc* represents the set of CHs and n is the number of nodes in a cluster.

The suggested k-medoids approach in Algorithm 3 provides phases for choosing a CH in a particular cluster. *K* and *N* are the inputs for Algorithm 3. This algorithm’s output includes *Mc*. The algorithm’s complexity is on average Θ(n). The algorithm’s outcome is an *Mc* set of the cluster heads each time.
**Algorithm3:** Cluster Head Selection.**Input:** *K*, *N***Output:** *M*  1.**Begin:**  2.**repeat:**  3.    **if** *N* = 1 **then**  4.     *CH_i_* ← *N_i_*  5.     *M_i_* ← *CH_i_*  6.     **else** **if** N > 2 **then**  7.      *CH_i_* ← median (*N*) using (5)  8.      *M_i_* ← *CH_i_*  9.    **end** **if**  10.**until**: *CH* is selected for *K* clusters  11.**end**

#### 3.2.4. Node Leaving a Cluster

The OPRP offers a planned method for member nodes to leave a cluster. This results from a member node changing its path or accelerating or decelerating. The nodes most likely to leave a cluster are edge nodes, which are found close to their boundaries. Even when the node is not an edge node, it transforms into one shortly before leaving. Every member node in OPRP maintains a threshold, ε, to keep itself connected to a specific cluster. When a node wants to leave its cluster, this threshold is evaluated concerning an immediate neighbor node. Once this threshold is reached, the member node communicates a leaving message to its CH, indicating that it is willing to leave the cluster. The CH announces its departure and deletes any entries related to that node from its list. OPRP suggests updating ε as follows for a node that wants to leave its cluster:

(6) ϵ=μ×Δ,

where Δ represents a fading component and *μ* is the node’s transmission range that contributes to the definition of *ε* for a node in terms of *μ*. As soon as a leaving node reaches the fading area, also known as *μ* − *ε*, which is located around the boundaries of a given node’s communication range, the leaving node begins to leave the associated cluster. Depending on the circumstances, the departing node may join another cluster or form its own cluster.

Algorithm 4 offers instructions for removing a node from a specific cluster, utilizing our improved k-medoids algorithm. If the departing node is a CH, it specifies the CH re-election criteria. In that algorithm, Nr stands for the randomly chosen node, which is chosen as a temporary CH upon an existing CH’s departure from its cluster. This method takes the inputs K and N, and its output is a notification of Ni’s departure. The algorithm’s complexity is on average Θ(n). The algorithm’s output is the Ni node leaving a notification each time.
**Algorithm 4:** Node Leaving.**Input:***K*, *N***Output:** Leaving notification of *N_i_*  1.**Begin:**  2.**repeat:**  3.    **if** *K* ≠ φ **then**  4.     **if** *η*(*N_i_*, *N_i_*_+1_) > ε or η(*N_i_*, *N_i_*_−1_) > ε **then**  5.      **if** *N_i_* *≡* *CH_i_* **then**  6.       *CH_i_* ← *N_i_*  7.        perform *CH* selection  8.       **else**  9.        *CH_i_* → *N_i_*  10.      **end** **if**  11.     **end** **if**  12.    **end** **if**  13. **end** **repeat**  14. **End**

#### 3.2.5. CH Re-Selection

It is also feasible that the leaving node is the CH when considering the earlier standard for node exit from a cluster. In this scenario, the CH temporarily hands over to *N_r_*, a node selected at random, gathering data on all of the member nodes and conducting CH election similarly to Section 3.2.3.

## 4. Optimal Path Discovery Phase

In this phase, we try to find an optimal path to disseminate the warning message. In this regard, we present two algorithms.

### 4.1. Warning Message Dissemination

We suggest Algorithm 5, which outlines the methods for transmitting warning messages. The algorithm accepts three inputs: *S,* *D*, and 𝜏, where *S* stands for the source node, *D* for the destination node, and 𝜏 for the time step. There are two scenarios where S aims to send a warning message to *D*: either *D* is near one hop or farther away than one hop from *S*. The message is immediately disseminated in the first scenario. Nevertheless, suppose D is more than one hop away. In that case, S uses the assistance of intermediate relay nodes to communicate to *D*. The result of Algorithm 5 is whether or not a warning message was successfully transmitted. The algorithm’s complexity is on average Θ(n). The algorithm’s output is the failure or success in disseminating a WM each time.
**Algorithm5:** Warning Message Dissemination.**Input:** *DS*, and 𝜏**Output:** Failure or Success in the dissemination of a WM  1.**Begin:**  2.Build *NG_s_* for *S*  3.  **loop**  4.   **if** *D* ∈ *NG_s_* **then**  5.    *S* *>>* *D*  6.    **exit**  7.   **else**  8.     identify *O_f_*  9.     *S* *>>* *O_f_*  10.     *S* ← *O_f_*  11.     Build *NG* for newly deputed *S* at 𝜏*_i_*  12.   **end** **if**  13.  **end** **loop**  14.**end**

### 4.2. Optimal Path Discovery

Paths for disseminating warning messages alter at each time step in bi-directional road traffic due to regular topological changes. A direction-based approach that considers the source and destination nodes’ relative positions might be more effective. To achieve this, we suggest Algorithm 6, which takes *D*, *S*, and *NG* as inputs. The algorithm uses the relative locations of S and *D* and their moving directions in addition to a distance parameter to determine the optimum path for warning messages to reach a specific destination node. The choice of the *O_f_* that is the optimal path towards the *D* is Algorithm 6′s output. The algorithm’s complexity is on average Θ(n). The algorithm’s output is *O_f_* the optimal path towards the destination node each time. Five different scenarios are considered to examine the importance of the extra parameters added by OPRP for enhancing routing performance. The following subsections provide more information on these circumstances, which cover every individual scenario concerning the location of the source and destination nodes on the bi-directional highway.
**Algorithm6:** Optimal Path Discovery.**Input:** *DS*, and *NG_s_***Output:** O*_f_* the optimal path towards the destination node  1.**Begin:**  2.  **for** *i* = 0 **to** *NG_s_* -1  3.    *Δ_i_*←(*NG_s_*, *D*)  4.    H_i_←(*NG_s_*, *S*)  5.     **if** *H(S*,*D)* = 1 **then**  6.      **if** S = Rear and *D* *=* Front node **then**  7.         *δ_i_* ← *Δ_i_*/*H_i_*  8.      **else**  9.         *δ_i_* ← *Δ_i_ H_i_*  10.       **end** **if**  11.      **else**  12.        **if** *S* and *D* move toward each other **then**  13.         *δ_i_*←*Δ_i_*/*H_i_*  14.        **else**  15.         *δ_i_* ← *Δ_i_ H_i_*  16.        **end** **if**  17.      **end** **if**  18.    **end** **for**  19. *O_f_*←Min (*δ*)  20. **return** *O_f_*  21.**end**

#### 4.2.1. Scenario 1

**Figure 4:**
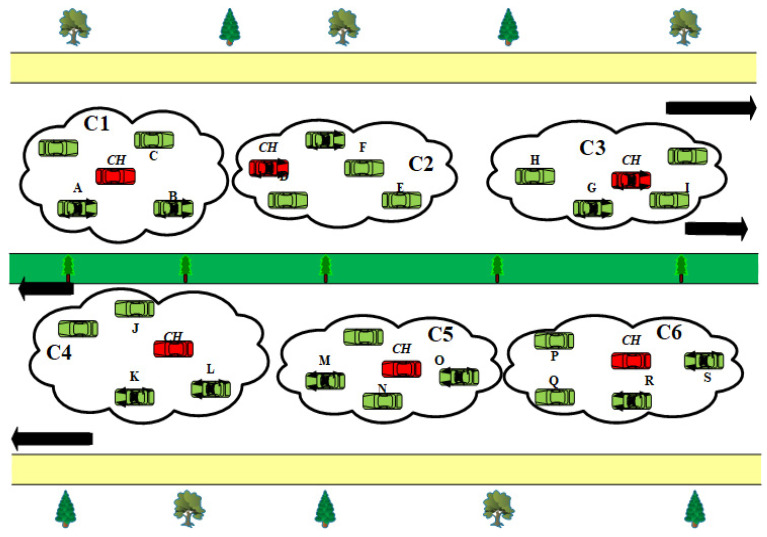
Dissemination of warning messages with different scenarios.

In this scenario, we consider a case revealed in Figure 4 with the source and destination nodes specified as follows:

- Source node C1: A is the rear node.
- Destination node C3: G is the front node.
- Source and destination nodes are moving in the same direction.

Assume there are two paths to reach from the source node C1: A to the destination node C3:G scenario mentioned above. The first path is the other side of a road to the source node, i.e., C1:A >> *CH* >> C4: *CH* *>>* C5: *CH* >> C6: *CH* >>C3: *CH* >> G, and the second path is C1: A >> *CH* >> C2: *CH* >> C3: *CH* >> G. Suppose cluster C4: *CH* is closer to the target than C2: *CH*, cluster C4: *CH* will be given preference for packet forwarding under the traditional greedy routing method, choosing the first path out of the available possibilities. Similarly, to this, direction-based techniques that choose a hop close to the source’s transmission range boundary in the direction of the destination will favor using the same node for the subsequent hop. In VANETs, these priority-based protocols cannot offer superior results when nodes move in various directions.

The number of hops on a path is influenced by the direction of nodes. It is not a sensible choice to choose a path with fewer hops on a given time step without considering the locations and the directions of nodes on the path because the number of hops on a path continues to be proportional to the delay in communication of WM. For instance, if we use the previously given paths to Node C3: G as an example, the first path, upon safe delivery of the WM, is C1: A >> *CH* >> C4: *CH* >> C5: *CH* >> C6: *CH* >> C2: *CH* >> C4: CH >> G. By the time a packet is received on C6: CH, the target Node C3: *CH* has moved out of its transmission range because the C6: CH is traveling in the opposite direction.

In order to get out of this predicament, Node C6: *CH* needs more intermediate relay nodes to enable the successful transmission of WMs. There are two options in such a situation:

- Cluster C6: *CH* does not find any forwarder further to reach the destination Node C3:G.
- Cluster C6: *CH* finds a forwarder C2: *CH* and goes into a path reconstruction process.

The case becomes exceedingly severe if no alternative forwarder is available because C6:*CH*, which moves in the opposite direction from the destination node, carries the packets with it, causing a message drop. Whenever a forwarder is still accessible in the second scenario, a path reconstruction procedure that adds additional nodes to the already specific path is started. On a newly built path, cluster C6: *CH* travels through additional cluster C2: *CH* to reach the destination cluster C3: *CH* >> G.

However, there is no change in the number of hops for the second path chosen by OPRP because all nodes travel in the same direction as the destination node. This shows clearly that the placement of intermediate relay nodes along a path significantly affects the prompt and accurate transmission of warning messages. When the source node is in the back and the destination node is in front, while both are going in the same direction, it is possible to grant such direction-based priority to routes if the ultimate distance for the following hop with the destination node is

(7) $$\delta_{i}=\frac{\Delta }{H\left(NG_{si\;}S\right)}$$

where *δ_i_* denotes the final distance of *NG_Si_* (i.e., the next hop to which the WM can be transmitted) from the destination node, Δ is the Euclidean distance between *NG_Si_* and the destination node, and 𝐻 (.) is the Hamming distance function.

The node with the lowest distance is calculated using Equation (7). That node will be favored as an *O_f_* over the other nodes. In our suggested protocol, if *H* *(S*, *D*) = 1, S stays behind D. Reducing the number of hops and delays during the transmission of WMs accomplishes our direction-based priority assignment process in the definition of the *O_f_* node. Reducing the number of hops and delays during the transmission of WMs completes our direction-based priority assignment process for the *O_f_* node. Reducing packet dropouts also increases the transmission dependability of these warning messages. Our suggested protocol is reliable because of this minimal delay and packet loss feature.

#### 4.2.2. Scenario 2

In this scenario, we take into consideration Figure 4 with the source and destination nodes specified as follows:

- Source node C3: G is the front node.
- Destination node C1: A is the rear node.
- Source and destination nodes are moving in the same direction.

Assume that there are two paths to reach the target Node C1: A in this scenario. The first path is the other side of a road to the source node, i.e., C3: G >> *CH* >> C6: *CH* >> C5: *CH* >> C4: *CH* >> C1: *CH* >> A, and the second path on the same side of the road is C3:G >> *CH* >> C2: *CH* >> C1: *CH* *>>* A. Here, the existing greedy protocols may send the WM to Node C2: *CH* rather than Node C6: *CH*. However, our suggested OPRP prefers Node C6: *CH* as the next hop. On successful message delivery, the ultimate path is C3: G >> *CH* >> C6: *CH* >> C1: *CH* >> A, since Node C6: CH travels in the opposite direction to the target node. This is due to the fact that by the time a WM is received by Node C6: *CH*, the destination node C1: *CH* >> A has entered its communication range, causing the message to be forwarded to Node C1: *CH* >> A immediately rather than using the originally intended path. We suggest Equation (8) for the selection of *O_f_* in this case, where *H* *(S*, *D)* = 1, and the source node stays in front of a destination node.

(8) $$\;\delta_{i}=\Delta \;H\left(S,\;NG_{s_{i}}\right)$$

where *δ_i_,* *Δ*, *S*, *NG_Si_*, and *H*(.), as described in Equation (1).

#### 4.2.3. Scenario 3

In this instance, we take into consideration the scenario shown in Figure 4 with the following requirements for the source and destination nodes:

- Source node C1: A is the rear node.
- Destination node C6: S is the front node.
- Source and destination nodes travel in opposite directions, toward each other.

This scenario is similar to Scenario 1 in terms of the positions of the source and destination nodes. However, the situation changes entirely because the two nodes are located on opposing sides of the road and going in opposite directions. The direction component is still essential in addition to the distance between nodes. Assume initially that there are two paths to go to the destination node C6:S in the scenario shown in Figure 4. The first path is C1: A >> CH >> C2: CH >> C3: CH >> C6: CH >> S, and the second path includes C1: A >> CH >> C4: CH >> C5: CH >> C6: CH >> S. The current greedy and direction-based algorithms will choose Node C4: CH as *Of*. based on fewer distances than Node C2: CH. However, in our suggested OPRP, Equation (7) is employed to establish the priority between nodes in NG_S_ when *H* *(S*, *D)* = 0 and the nodes are moving near one another. Therefore, Node C2: CH is preferred by OPRP as the *Of*. Hence, the final path becomes C1: A >> CH >> C2: CH >> C6: CH >> S, as the destination Node C6: S and the source Node C1: A are traveling towards each other by the time WM reaches the C2: CH communication range.

#### 4.2.4. Scenario 4

In this instance, we take into consideration a scenario shown in Figure 4 with the following requirements for the source and destination nodes:

- Source node C3: G is the front node.
- Destination node C4: K is the rear node.
- Source and destination nodes travel in opposite directions, away from each other.

Regarding where the destination and source nodes are located on the road, this scenario is similar to Scenario 3. However, the issue is still difficult to handle because, in this scenario, the source and destination nodes’ directions are opposite. Here, assume that there are two possible paths to reach the destination node C4: K from Node C3: G—CH >> C6: CH >> C5: CH >> C4: CH >> K and C3: G >> CH >> C3: CH >> C1: CH >> C4: CH >> S. Again, the traditional greedy protocols will choose Node C2: CH as *O_f_*, but as the nodes move, the chosen path will result in more hops than the other path. This is due to the fact that the destination node has left the communication range of the warning message by the time it reaches Node C1: CH. Therefore, a path reconstruction occurs, and the packets are routed on the new path. The situation is more complicated when Node C cannot find a node to forward the message. As a result, our suggested protocol prioritizes Node C6: CH by employing Equation (8). When *H* *(S*, *D)* = 0 and the destination and source move apart, the WM is transmitted through the second feasible path.

#### 4.2.5. Scenario 5

In this instance, we take into consideration the scenario shown in Figure 4 with the following requirements for the source and destination nodes:

- Source node C6: S is the rear node.
- Destination node C1: A is the front node.
- Source and destination nodes travel in opposite directions, toward each other.

This scenario is similar to Scenario 3 in terms of the positions of the source and destination nodes. However, the situation changes entirely because the two nodes are located on opposite sides of the road and travel in opposite directions, toward each other. The directional component is still essential in addition to the distance between nodes. Assume initially that there are two paths to go to the destination node C1: A in the scenario shown in Figure 4. The first path is C6: S >> CH >> C3: CH >> C2: CH >> C1: CH >> A, and the second, C6: S >> CH >> C5: CH >> C4: CH >> C1: CH >> A. The traditional greedy protocol selects the first path as *O_f_*. However, in our suggested OPRP, Equation (7) is employed to establish the priority between nodes in NG_S_ when *H* *(S*, *D)* = 0 and the nodes are moving near one another. Therefore, Node C5:CH is preferred by OPRP as the *Of*.

## 5. Performance Evaluation

The effectiveness of our suggested OPRP procedure is assessed in this section concerning DABFS [8] and ID-LAR [7]. We employed the Mobility Model Generator for Vehicular Networks (MOVE) [35], Simulation of Urban Mobility (SUMO) [36], and ns-2.35 to assess performance in a realistic vehicular environment. Route information, intersections, and node positions were produced by SUMO and MOVE and used by ns-2.35. Unless otherwise stated, all simulations were based on Scenarios 1 to 5, depicted in Figure 4 in Section 4. The performance evaluation parameters for the protocols mentioned above are listed in Table 3. Nodes with omni-directional antennas are dispersed at random. The nodes were moving bi-directional at varying rates belonging to a set, χ, with lower and upper bounds of 0 m/s and 42 m/s, respectively. The acceleration or deceleration that nodes achieved was in the range of 1 to 6 m/s^2^. In addition, Table 4 shows the number of nodes on the highway was categorized as sparse, medium, or dense. This classification adjusts the node density by actual traffic [37].

**Figure 5:**
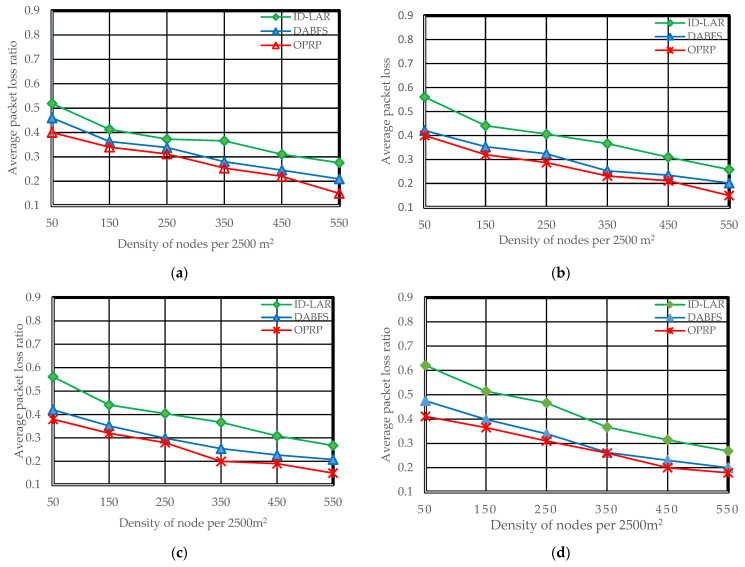

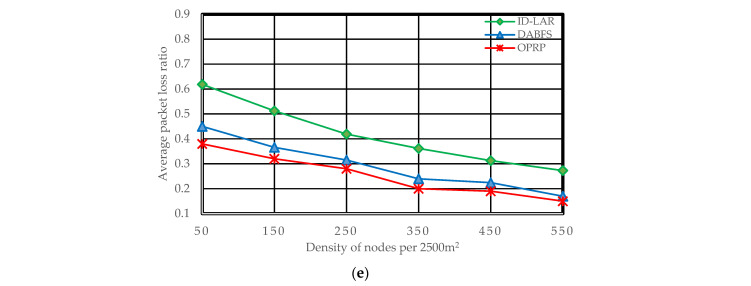
Average packet loss rate during the dissemination of WM. (**a**) Scenario 1: *H* *(S,* *D)* = 1, *S* is rear and *D* is front node. (**b**) Scenario 2: *H* *(S,* *D)* = 1, *S* is front and *D* rear. (**c**) Scenario 3: *H* *(S,* *D)* = 0, *S* and *D* are moving toward each other. (**d**) Scenario 4: *H* *(S,* *D)* = 0, *S* and *D* are moving away from each other.(**e**) Scenario 5: *H(S, D)* = 0, *S* and *D* are moving toward each other.

**Figure 6:**
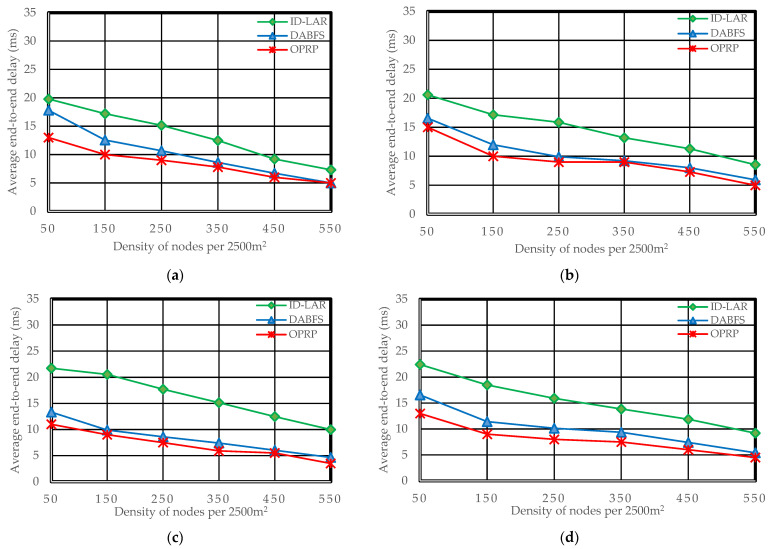

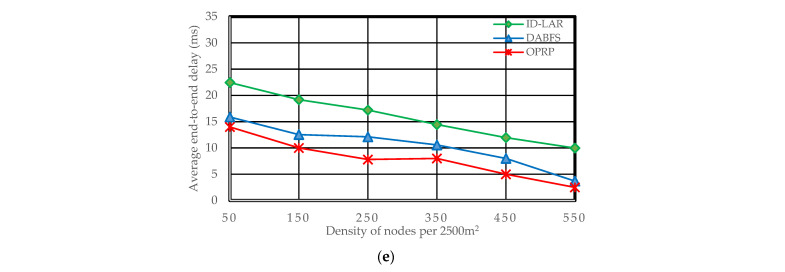
End-to-end delay during the dissemination of WM. (**a**) Scenario 1: *H* *(S,* *D)* = 1, *S* is rear and *D* front node. (**b**) Scenario 2: *H* *(S,* *D)* = 1, *S* is font and *D* is rear node. (**c**) Scenario 3: *H* *(S,* *D)* = 0, *S* and *D* are moving toward each other. (**d**) Scenario 4: *H* *(S,* *D)* = 0, *S* and *D* are moving away from each other. (**e**) Scenario 5: *H* *(S,* *D)* = 0, *S* and *D* are moving toward each other in opposite directions.

**Figure 7:**
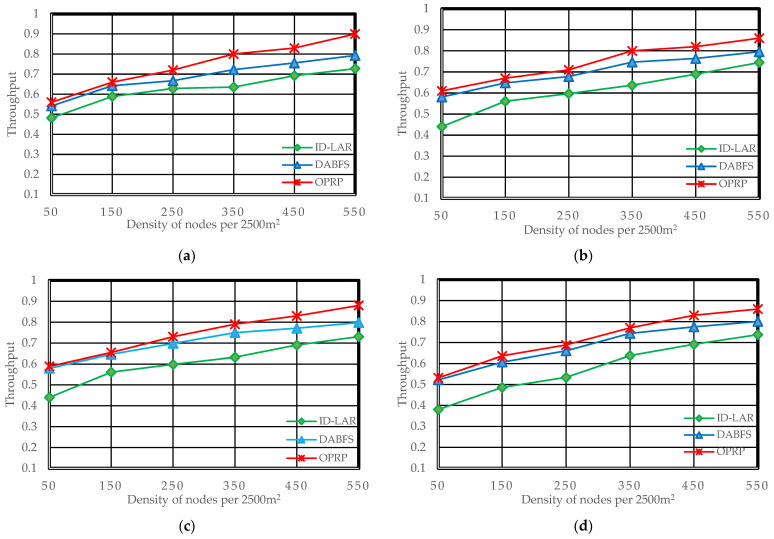

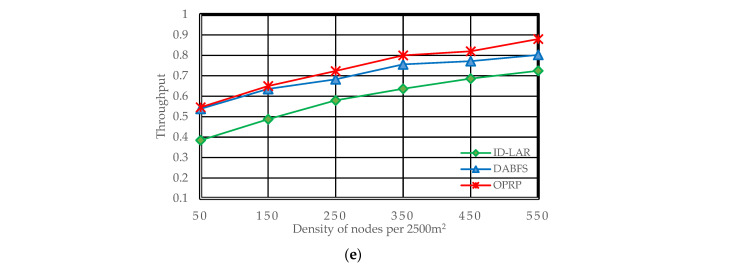
Throughput during the dissemination of WM. (**a**) Scenario 1: *H* *(S,* *D)* = 1, *S* is rear, and *D* is front. (**b**) Scenario 2: *H* *(S,* *D)* = 1, *S* is front, and *D* is rear. (**c**) Scenario 3: *H* *(S,* *D)* *=* 0, *S* and *D* are moving toward each other. (**d**) Scenario 4: *H* *(S,* *D)* *=* 0, *S* and *D* are moving away from each other. (**e**) Scenario 5: *H* *(S,* *D)* *=* 0, *S* and *D* are moving toward each other in opposite directions.

Throughput (Tr), packet loss rate (Pr), and end-to-end delay (Er), which are frequently used metrics in state-of-the-art to analyze VANET routing protocols for efficient distribution of WMs with minimal latencies and few packet losses, were among the efficiency evaluation measures [38,39]. Figure 5, Figure 6 and Figure 7 illustrate the simulation results for each metric. The Figure 7a–e in each figure correspond to the five different scenarios presented in Scenarios 1 through 5 of Section 4.2.

### 5.1. Packet Loss Rate

This parameter is the ratio of dropped packets, which may be calculated as follows:

(9) $$P_{r}=\frac{\Sigma_{i=1}^{p_{l}}P_{l}{}_{i\;\;}}{P_{t}},$$

where *Pr* denotes the packet loss rate, *Pli* is a dropped packet, and *Pt* is the total number of packets delivered across the network.

Due to the repeated network changes, the path choice during WM transmission remains crucial. In VANETs, topological changes are frequently caused by fast nodes traveling in various directions. The nodes continuously move, enter, and exit each other’s communication ranges. As a result, paths are frequently dropped and new ones generated, potentially leading to network partitions. As a greater number of nodes enhances network connectivity and lowers packet drops, the probability of such network partitioning seems higher in scatter networks than in dense networks. The packet loss ratio decreases as the density increases because nodes are closer. Hence, the proposed protocol with clustering and the compared protocols without clustering becomes more intimate in the results. The three protocols’ results illustrated in Figure 5a–e reveal such behavior.

### 5.2. End-to-End Delay

End-to-end delays can be computed as:

(10) $$E_{r}=\frac{\Sigma_{i=1}^{R_{p}}E_{d_{i}\;}}{R_{p}},\;{\;}_{}$$

where the terms *Er* and *E_di_* and *Rp* stand for the end-to-end delay ratio, the individual packet delay, and the total number of packets received. Due to the increased number of hops in DABFS and ID-LAR, as a result of the path reconstruction process, as detailed in Section 4.2.1, the total number of send and receive operations also increases. The efficiency of both DABFS and ID-LAR is negatively impacted by the growing number of transmitting and receive operations because they are time-consuming. However, by choosing the next hops traveling in the target node’s direction, OPRP reduces this latency. The end-to-end delay decreases as the density increases because nodes are closer. Hence, the proposed protocol with clustering and the compared protocols without clustering become clearer in the results. Figure 6a–e reveals Scenario 5.

### 5.3. Throughput

This parameter, which can be calculated, refers to the proportion of packets received on the destination nodes of all packages sent by the source nodes.

(11) $$T_{r}=\frac{\Sigma_{i=1}^{R_{p}}R_{p_{i}\;}}{P_{t}},{\;}_{}$$

where $T_{r}$ refers to the network throughput gain, $P_{t}$ refers to the total number of packets sent by the source node, and $R_{p_{i}\;}$ denotes a specific packet received by a target node.

As it counts the number of packets successfully delivered to target nodes, throughput is a crucial metric for performance evaluation. End-to-end delays and packet losses impact the throughput of the network. Figure 7a,b show improved throughput of OPRP compared to DABFS and ID-LAR due to our original direction-based path selection. Additionally, OPRP follows the same pattern under challenging circumstances where the source and destination are on the opposite sides of the road by significantly improving throughput, as demonstrated in Figure 7c,d. Additionally, as shown in Figure 7e, OPRP outperformed the other protocols, improving throughput restoration state packet forwarding.

Moreover, the packet loss ratio and end-to-end delay decrease as the density increases because nodes are closer. As a result, the throughput increases. Therefore, the proposed protocol with clustering and the compared protocols without clustering are clearly illustrated in the results.

## 6. Conclusions and Future Work

Dissemination of WMs is the primary method used in vehicular accident avoidance. Collisions between vehicles may result from delays or packet drops while disseminating WMs. In order to address the complex nature of bi-directional highway settings and offer effective path selection, we have developed an optimal path routing protocol (OPRP) that considers two metrics in addition to the distance metric. A node’s movement direction is included in the first parameter based on Hamming distance, and the source and destination nodes’ relative locations are included in the second parameter. We showed the importance of these variables in determining the next hop in bi-directional road traffic. This work has shown that the mentioned parameters can route WMs to increase throughput, decrease packet loss and delay, and accommodate topological changes in the transmission process. OPRP significantly outperforms other leading VANET routing protocols, according to simulation results. Extension of OPRP without clustering for the urban scenario with GPS-less localization will be part of our future study. Determining how channel conditions affect the transmission of WMs and secure communication can also be considered in future work. An SDN-based warning message dissemination using machine learning and deep learning can also be considered in future work.

## Figures and Tables

**Table 1:** Comparison of VANET WM dissemination techniques.

Technique	Year	Consider Bi-Directional Traffic	Network Scenario	WM Dissemination Mechanism	Clustering-Based	PDR	Delay
DVCAST [15]	2010	✕	Highway	Broadcast, SCF	✓	Medium	High
Schwartz et al. [16]	2011	✕	Highway	Broadcast, SCF	✓	High	Medium
Chen et al. [17]	2014	✕	Highway	Broadcast, SCF	✓	Medium	Medium
Nguyen et al. [18]	2017	✕	Highway	SCF	✓	Medium	Medium
Yaqoob et al. [9]	2018	✕	Urban	Fog server-based	✓	Medium	Medium
DABFS [8]	2019	✓	Highway	Direction-based	✓	High	Low
Kamakshi et al. [19]	2019	✕	Highway	Broadcast	✓	Medium	Medium
TBEMD [14]	2019	✕	Urban	Broadcast	✓	Medium	Medium
RBO-EM [32]	2020	✕	Highway	Broadcast, uni-cast	✓	High	Medium
P-DACCA [22]	2020	✓	Highway	Broadcast	✓	Medium	Low
EEMDS [31]	2021	✕	Urban	Broadcast, uni-cast	✓	High	Low
BURP [33]	2022	✓	Urban	Broadcast	✓	High	Low
OPRP		✓	Highway	Direction-based	✓	High	Low

**Table 2:** List of notations.

Notation	Description
>>	A packet transmitted from left to right node
*Ack*	*Hello* packet received response acknowledgment
*O_f_*	Optimal forwarder
*x*	Set of speed for nodes
*CNP*	Current position of a node
*D*	Destination node
*δ*	Final distance between *NG_i_* and *D*
*Δ*	Distance between *NG_i_* and *D*
*E_p_*	End-to-end delay for packet
*E_r_*	End-to-end delay ratio
*η*	Euclidean distance
*κ*	Hamming distance
*n*	A member node
*N*	Set of all nodes
*NG*	Neighboring table for a node
*NID*	Node identity
*P_l_*	Packet dropped
*Pr*	Packet loses rate
*Pt*	Total packet transmitted across the network
*R_p_*	Total received packet
*S*	Source node
τ	Timestamp for the last received *ACK*
*T_r_*	Network throughput
ɤ	The direction of the node

**Table 3:** List of parameters.

Parameter	Values
Simulation area	5000 m^2^
Traffic type	Bi-directional highway traffic
Number of Nodes	0–500
Speed of nodes χ	0 m/s–42 m/s
Acceleration/Deceleration attained by nodes	1 m/s^2^–6 m/s^2^
Transmission range of nodes	150 m
*Hello* packet interval	1 s
Simulation time	300 s

**Table 4:** Categorization of nodes for density.

Network Type	Number of Nodes per 2500 m^2^
Sparse	001<=*N*<=200
Medium	201<=*N*<=400
Dense	401<=*N*<=500
